# Outcomes of cases with complete hydatidiform mole coexisting with a fetus: a single-center study

**DOI:** 10.1007/s10147-025-02815-0

**Published:** 2025-06-28

**Authors:** Kaoru Niimi, Mayu Shibata, Kosuke Yoshida, Eiko Yamamoto, Seiji Sumigama, Yuko Yasui, Yuki Nishiko, Kimihiro Nishino, Hiroaki Kajiyama

**Affiliations:** 1 Department of Obstetrics and Gynecology, Nagoya University Graduate School of Medicine, 65 Tsurumai-Cho, Showa-Ku, Nagoya, 466-8550 Japan; 2 Department of Healthcare Administration, Nagoya University Graduate School of Medicine, Nagoya, Japan; 3 Office of International Affairs/International Medical Education, Nagoya University Graduate School of Medicine, Nagoya, Japan

**Keywords:** CHMCF, Low-risk GTN, Post-molar GTN

## Abstract

**Background:**

Complete hydatidiform moles coexisting with a fetus (CHMCF) are uncommon. Although CHMCF is associated with perinatal complications and post-molar gestational trophoblastic neoplasia (GTN), necessitating post-delivery chemotherapy, live birth remains feasible. This report presents 14 cases of CHMCF in Japan.

**Methods:**

We reviewed medical records of patients with CHMCF treated at our hospital from 2000 to 2020 and summarized clinical data, including maternal age, pregnancy details, delivery outcomes, fertility treatments, serum human chorionic gonadotropin (hCG) levels, and ultrasonography findings.

**Results:**

Fourteen cases of CHMCF were diagnosed. The average age of the mothers was 30.6 years, with the majority conceiving following fertility treatment. The mean gestational age at diagnosis was 12 weeks. Six patients maintained their pregnancies, leading to two live births through emergency cesarean section. Eight patients exhibited spontaneous regression following treatment and pregnancy interruption, achieving negative serum hCG levels within 17.4 weeks. Six patients experienced post-molar GTN, including the two who had live births. One patient presented with FIGO stage I disease, while five patients had stage III lung metastases. All patients received chemotherapy, averaging nine courses, achieving remission within 13.7 weeks.

**Conclusion:**

The occurrence of GTN was higher after CHMCF than after typical complete hydatidiform moles. Despite the heightened risk of premature birth, some patients with CHMCF who maintain their pregnancies can successfully deliver live babies. Informed consent is essential for patients with CHMCF when considering pregnancy continuation. A team approach involving gynecological oncologists, obstetricians, and neonatologists is essential for effective diagnosis and treatment.

## Introduction

The concurrent identification of a coexisting fetus and hydatidiform mole in utero during early pregnancy can suggest two potential conditions: a partial hydatidiform mole or a twin pregnancy comprising a hydatidiform mole alongside a fetus. A complete hydatidiform mole coexisting with a fetus (CHMCF) is a rare occurrence, with an estimated incidence of one case per 22,000–100,000 pregnancies [1]. Management of partial moles is rarely problematic, as the majority are triploid and often lead to intrauterine fetal death by mid-pregnancy. Fetuses diagnosed with CHMCF have normal chromosomes, indicating the potential for a successful live birth. However, these pregnancies are associated with a significantly increased risk of perinatal complications, including spontaneous abortion, intrauterine death, gestational hypertension, uterine bleeding, and preeclampsia [2]. Further, many pregnancies, if continued, lead to post-molar gestational trophoblastic neoplasia (GTN) [3]. In cases of post-molar GTN, such as an invasive mole, chemotherapy is necessary if diagnosed following delivery. The diagnosis of CHMCF in patients and children complicates the decision-making regarding the continuation or termination of a pregnancy with a coexisting mole, given the associated risks.

Due to the scarcity of CHMCF in Japan, we present 12 cases of CHMCF diagnosed at our institution to address this knowledge gap. Herein, we describe the clinical course of CHMCF, focusing on the interruption strategy, incidence of post-molar GTN, and the outcome of subsequent pregnancies.

## Patients and methods

### Retrospective study flow

We conducted a retrospective review of the medical records of patients diagnosed with CHMCF at our hospital from 2000 to 2020. Patients suspected of having CHMCF from hospitals or clinics in Aichi and neighboring regions were referred to our hospital. Case 4 was previously reported by our group as a case report [4].

All patients underwent ultrasonography and serum human chorionic gonadotropin (hCG) measurement. Clinical diagnoses were made by oncologists and perinatologists, and cases were included in the study if they met the criteria for CHMCF. Data collection encompassed patient demographics, diagnostic findings, treatment courses, and pregnancy outcomes.

### Clinical diagnostic criteria

The clinical diagnosis of CHMCF was established based on a combination of imaging findings and biochemical markers. Ultrasonography was used to identify the presence of a coexistent hydatidiform mole and a surviving fetus. Serum hCG levels were measured to assess abnormal trophoblastic activity. Amniotic fluid testing was proposed to confirm that the karyotype of the surviving fetus was normal in patients who continued their pregnancies.

### CHMCF diagnostic procedures

Histopathological examination served as the gold standard for the final diagnosis of CHMCF. In addition to routine histological evaluation, selected cases underwent molecular diagnostic procedures, including short tandem repeat (STR) analysis, to distinguish CHMCF from other molar pregnancies or placental mosaicism. p57KIP2 immunostaining was applied in all cases for differential diagnosis between complete hydatidiform mole (CHM) and partial hydatidiform mole (PHM).

### Clinical data collection parameters

For all patients, we collected data on maternal age, gravidity, parity, history of fertility treatment, initial serum hCG levels, and ultrasonographic findings at diagnosis. In cases that progressed to live birth, we documented the course of pregnancy, delivery outcomes, and neonatal status. For patients who underwent termination, details regarding the method of termination, need for blood transfusion, and subsequent follow-up were recorded. In post-molar GTN cases, we analyzed initial hCG levels, timing of treatment initiation, presence of metastases, chemotherapy regimens, time to hCG normalization, total number of treatment cycles, and treatment-related side effects.

### General management protocols

Patients who elected to terminate pregnancy underwent procedures based on gestational age and clinical conditions. In the first trimester, suction curettage was the preferred method of termination. For mid-trimester terminations, vaginal delivery was induced using laminaria for cervical dilation, followed by prostaglandin-based vaginal agents. In all cases, including vaginal delivery and cesarean section, curettage was performed during terminations. The number of curettages was also examined. Hemorrhage management strategies included prophylactic uterotonics and transfusions, as necessary.

### GTN treatment approach

In cases of post-molar GTN, chemotherapy regimens evolved over the study period. Until 2011, the first-line chemotherapy regimen consisted of methotrexate and actinomycin D (MA therapy). Methotrexate (20 mg) was administered via intramuscular injection, and actinomycin D (0.5 mg) via intravenous injection, both given once daily for four consecutive days within a 14-day cycle. The second-line regimen involved etoposide and actinomycin D, with etoposide (100 mg) and actinomycin D (0.5 mg) administered intravenously over a similar four-day cycle. Since 2011, the preferred regimen has been five-day methotrexate therapy, consisting of methotrexate (20 mg) administered intramuscularly once daily for five consecutive days within a 14-day cycle. The alternative option remained actinomycin D (0.5 mg), administered intravenously once daily for five consecutive days within the same cycle. Chemotherapy was continued until serum hCG levels dropped below 0.5 mIU/mL, followed by 2–3 additional cycles to ensure remission. Follow-up for post-molar GTN extended to five years, with documentation of long-term outcomes, including subsequent pregnancies and births.

### Ethical considerations

This study was approved by the institutional review board of Nagoya University Graduate School of Medicine (approval number: 2019-0106). Given its retrospective nature, informed consent was obtained using the opt-out method, allowing patients to decline participation through publicly accessible information. Patient confidentiality was maintained, and all data were anonymized before analysis.

## Results

Between 2000 and 2020, our hospital diagnosed and treated 14 cases of CHMCF. Table 1 presents the clinical course of each patient. The mean age of the patients at the time of diagnosis was 30.6 years. Ten patients became pregnant following receiving fertility treatment, including seven who utilized fertility medications, one who underwent artificial insemination, and two who underwent in vitro fertilization. The mean gestational age at CHMCF diagnosis was 12 weeks (range 8–25). The mean serum hCG level at diagnosis was 519,746 mIU/mL (16,000–9,098,068 mIU/mL). In six cases, serum hCG levels exceeded 1,000,000 mIU/mL prior to delivery and pregnancy termination. The serum hCG levels of patients who did not develop post-molar GTN (Cases 1–8) were negative, averaging 17.4 weeks (range 11–29 weeks). After confirming negative serum hCG levels, all patients maintained negative serum hCG levels. The amount of hemorrhage could not be accurately assessed because the counts included hydatidiform moles and fetal parts. Only Case 12 required a blood transfusion. Further, ovarian enlargement of more than 6 cm was observed in Cases 2 and 8.

**Table 1:** Clinical course of 14 cases of CHMCF

Case	Age	Fertility treatment	hCG at diagnosis (mIU/mL)	GA at diagnosis (wk)	maximum hCG (mIU/mL)	GA at maximum hCG (wk)	Desire to continue pregnancy	hCG Before termination (mIU/mL)	GA at termination (wk)	Pregnancy outcome/ Termination method	STR analysis	Post-molar GTN	Priod to negative hCG after termination (wk)	
1	32	CC + hMG	360,450	8	553,161	10	–	553,161	10	AA/ D&C 1	–	–	17	
2	28	IVF-ET 2 eggs	242,411	9	329,286	9	+	329,286	10	SA/ D&C 2	–	–	14	
3	27	CC + hMG + AIH	226,935	9	232,525	10	–	232,525	10	AA/ D&C 2	–	–	29	
4	37	CC	218,800	10	218,800	10	–	201,732	12	AA/ D&C 2	+	–	15	
5	25	-	9,098,068	11	9,098,068	11	–	9,098,068	11	AA/ D&C 1	–	–	17	
6	22	-	257,711	12	257,711	12	–	190,934	14	AA/ VD + D&C 1	+	–	11	
7	34	CC	16,000	12	1,000,000	15	–	1,000,000	15	AA/ VD + D&C 2	+	–	NA	
8	29	-	1512,289	13	1,804,202	16	+	1,804,202	16	SB/ VD + D&C 1	–	–	19	
9	25	CC + hMG	572,995	9	572,995	9	–	572,995	10	AA/ D&C 2	–	+	–	
10	37	-	466,496	10	466,496	10	+	445,279	10	SA/ D&C 2	+	+	–	
11	39	IVF-ET 2 eggs	679,111	12	1,401,541	13	–	1,401,541	14	SB/ VD + D&C 2	+	+	–	
12	31	CC	754,360	13	3,518,102	19	+	1,303,782	24	live birth/ CS + D&C 1	–	+	–	
13	36	AIH	783,497	14	1,908,752	15	+	1,046,789	16	SB/ VD + D&C 1	–	+	–	
14	27	hMG + hCG	666,165	25	674,950	26	+	415,804	29	live birth/ CS + D&C 1	–	+	–	

*AA* artificial abortion, *SA* spontaneous abortion, *SB* stillbirth, *VD* vaginal delivery, *CS* cesarean section, *D&C* dilatation and curettage, *CC* clomiphene citrate, *IVF* in vitro fertilization, *ET* embryo transfer, *hMG* human menopausal gonadotropin, *hCG* human chorionic gonadotropin, *AIH* artificial insemination by husband, *STR* short tandem repeat, *GTN* gestational trophoblastic neoplasia, *GA* gestational age, *wk* week, *NA* not available

Six patients (cases 2, 8, 10, 12, 13, and 14) expressed a desire to continue their pregnancies after receiving a diagnosis of CHMCF. However, case 13 was diagnosed with lung metastasis at 16 weeks and ultimately chose to terminate the pregnancy, while cases 2, 8, 10, and 13 experienced intrauterine fetal death at 10, 16, 10, and 16 weeks, respectively. Eight additional patients expressed a desire to terminate their pregnancies following the initial diagnosis. Finally, two patients (Cases 12 and 14) delivered live infants through emergency cesarean sections at 24 and 29 weeks of gestation, respectively. Patient 12 was hospitalized for urgent preterm labor and developed severe anemia due to tumor hemorrhage, requiring emergency cesarean section. She received a transfusion of red blood cells and fresh frozen plasma due to significant hemorrhage (4,584 g including molar tissues). Patient 14 received an emergency cesarean section due to preterm premature rupture of membranes. Both infants exhibited no apparent physical deformities and developed without significant developmental issues.

**Fig. 1:**
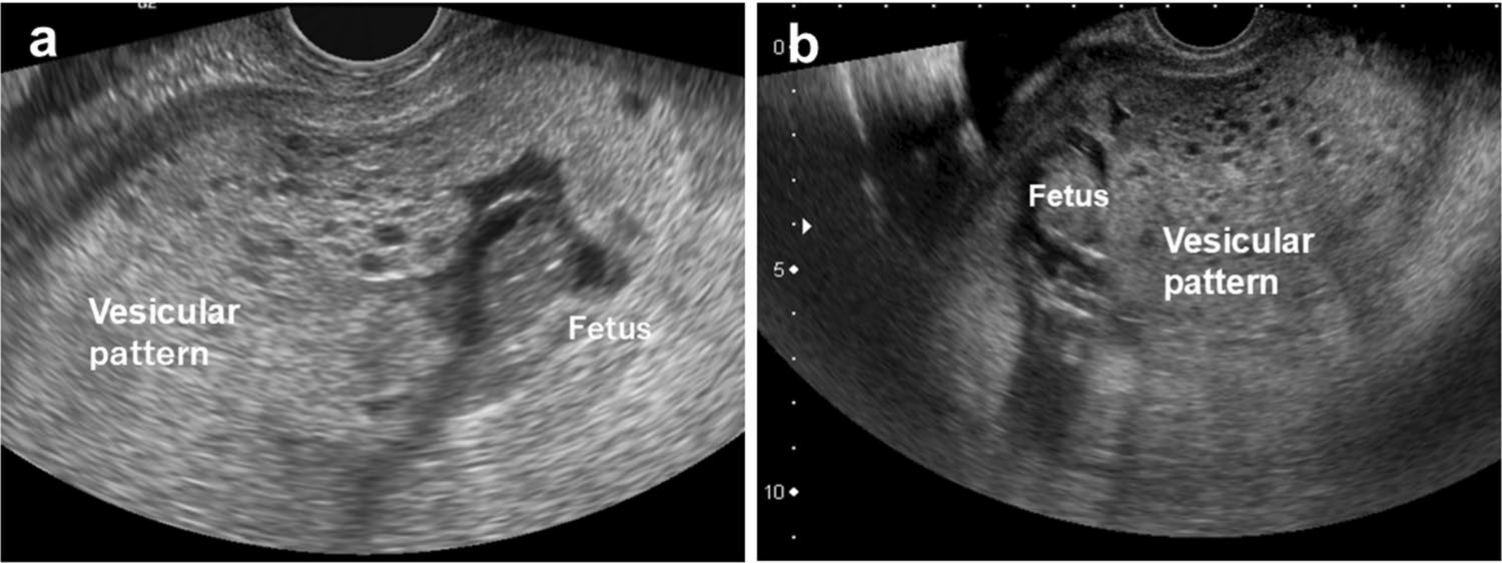
Ultrasound findings. Ultrasound images of cases 2 (**a**) and 9 (**b**), revealing a cystic mole and a portion of the fetus in both cases

**Fig. 2:**
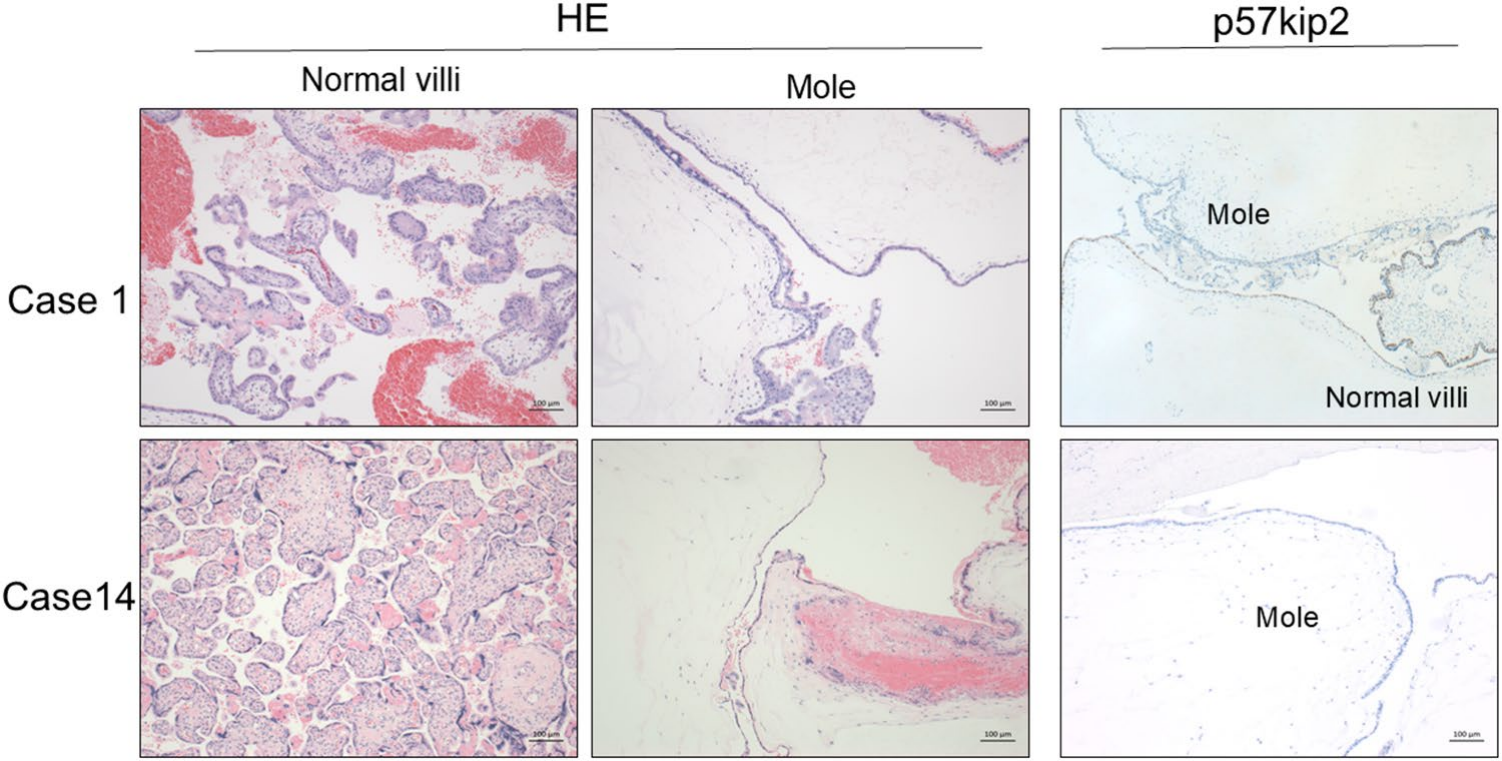
Pathological findings. Hematoxylin–eosin staining of normal villi and moles, and p57kip2 staining in Case 1 (top) and Case 14 (bottom). p57kip2 staining showing a mixture of positive normal villi and negative molar areas in Case 1. Case 14 showed only a p57-negative molar area

A clinical diagnosis of fetal coexistence was established in all cases based on ultrasound findings and serum hCG levels. All cases exhibited typical ultrasound findings, demonstrating a clear demarcation between normal placental tissue and vesicular chorionic villi (Fig. 1). Pathological examination demonstrated a combination of normal chorionic villi, placenta, and molar components in each case (Fig. 2). Consequently, all cases of CHMCF were diagnosed based on pathological findings. p57kip2 immunochemistry effectively identified partial or complete hydatidiform moles (Fig. 2, right). STR analysis was conducted in five cases, revealing coexisting complete hydatidiform moles and fetuses originating from a single sperm-fertilized homozygous mole and a mole with normal parental karyotypes.

Seven out of the 14 patients exhibited spontaneous regression following treatment and pregnancy interruption, whereas six patients developed subsequent post-molar GTN, including two who achieved live births. We then compared patients diagnosed with and without GTN (Table 2). The clinical characteristics, including maternal age, gravidity, parity, gestational age at diagnosis, serum hCG level at diagnosis, maximum hCG levels, and pregnancy outcomes, exhibited similarities. The median gestational age at pregnancy termination did not exhibit significant differences between patients with GTN and those without (15 and 11.5 weeks, respectively). Table 3 illustrates the characteristics of patients diagnosed with post-molar GTN. One patient presented with FIGO stage I disease, while five patients exhibited stage III lung metastases. All patients underwent chemotherapy, resulting in remission. The average number of courses of chemotherapy administered was 9 (range 7–13 courses). Serum hCG levels became negative at an average of 13.7 weeks, with a range of 6–20 weeks post-treatment initiation.

**Table 2:** Comparison of patients diagnosed with GTN vs. those without

Clinical characteristics	With GTN *n* = 6	Without GTN *n* = 8	*p* value
Maternal age (mean)	32.5	29.3	0.398
Gravidity (median)	1	1	0.431
Parity (median)	0	0	0.845
Fertility treatment, *n* (%)	5 (83%)	5 (63%)	0.416
Gestational age at diagnosis, wk (median)	12.5	10.5	0.223
Serum hCG level at diagnosis, mIU/mL (median)	672,638	250,061	0.452
Maximum hCG levels, mIU/mL (median)	1,038,246	441,224	0.828
Gestational age at termination of pregnancy, wk (median)	15	11.5	0.187
Pregnancy outcomes			
Abortion or still birth, *n* (%)	4 (67%)	8 (100%)	0.175
Live birth, *n* (%)	2 (33%)	0 (0%)	0.175

*GTN* gestational trophoblastic neoplasia, *hCG* human chorionic gonadotropin, *wk* week

**Table 3:** Characteristics of patients with post-molar GTN

Case	GA at pregnancy termination (wk)	Pregnancy outcome	FIGO stage	Chemo therapy	Total courses	hCG negative (wk)	Subsequent pregnancies	Follow up period after chemotherapy (month)
9	10	Abortion	I	MA/EA/ActD	7	12	Vaginal delivery	72
10	10	Abortion	III	MTX/ActD	9	16	None	12
11	14	Still birth/ Vaginal delivery	III	MA	6	6	Vaginal delivery	32
12	24	Caesarian section	III	MTX/ActD	9	12	None	16
13	16	Still birth/ Vaginal delivery	III	MTX	10	16	Vaginal delivery	16
14	29	Caesarean section	III	MA/EA	13	20	Caesarian section	68

*GTN* gestational trophoblastic neoplasia, *hCG* human chorionic gonadotropin, *wk* week, *MA* methotrexate, *EA* etoposide, actinomycin D

No recurrences were observed during the average follow-up period of 39.3 months (16–68 months) following the remission of hydatidiform mole or post-molar GTN. Ultimately, 11 of the patients successfully delivered live children.

## Discussion

In the present study, we examined the clinical progression of CHMCF diagnosed at our institution. Overall, 6 of 14 patients with CHMCF expressed their desire to continue their pregnancy following informed consent obtained from gynecologic oncologists and perinatologists. Thirty-three percent of the patients successfully maintained gestation and achieved live births. This outcome was comparable to earlier findings indicating that the rate of live births among women who opted against pregnancy interruption was 38% [5]. Wang et al. previously indicated that the average delivery time for cases with potential live births was 34.3 weeks, whereas the live birth rate across all CHMCF, including those opting for termination, increased from 16.7% in 2000 to 50% in 2017 [6]. The reported preterm birth rate was 49. 5% (range 25–73.1%). At our institution, the gestation ages at delivery for two live births were 24 and 29 weeks, respectively. Despite the limited number of cases, preterm birth appeared to be prevalent in CHMCF. Hemorrhaging during delivery is the predominant complication of CHMCF during pregnancy, followed by hypertension, preeclampsia, and hyperthyroidism [2, 7]. Controlling these complications in the uterine molar tissues is challenging. Our patient, who gave birth at 24 weeks gestation, experienced severe anemia resulting from uncontrolled hemorrhaging. Although our two delivery cases underwent cesarean section, previous research indicates that the mode of delivery does not correlate with subsequent disease following molar pregnancy in patients with CHMCF [6]. Consequently, the method of childbirth should be evaluated according to obstetric accommodations.

In the present study, six of 14 (42.9%) CHMCF cases developed subsequent post-molar GTN. Previous reports indicate that 10–20% of complete hydatidiform moles and 0.5–4% of partial hydatidiform moles progress to post-molar GTN [8]. Additionally, an average of 35.9% (19.0–47.0%) of CHMCF cases resulted in subsequent GTN. These results suggest a higher incidence of subsequent GTN in patients with CHMCF than those with typical complete moles. The median numbers of gestational weeks in the six cases with GTN and the eight cases without GTN were 15 and 11.5 weeks, respectively, with no significant difference observed. Several studies have similarly found no association between gestational weeks and the incidence of subsequent GTN after CHMCF [9–12], which aligns with the result of the present study.

In the present study, one patient developed lung metastases during pregnancy. This agrees with a previous report of a case of CHMCF that progressed to choriocarcinoma during pregnancy [13]. Thus, patients with CHMCF who continue their pregnancy require a rigorous follow-up protocol that includes chest radiography and measurement of hCG value assessment during pregnancy. Most cases of post-molar GTN cases followed by CHMCF attain complete remission through common low-risk GTN treatments, including single-agent chemotherapy or hysterectomy. All six patients with post-molar GTN achieved remission with single- or multiple-agent chemotherapy without any recurrence. All 11 patients desiring to have children ultimately achieved live births, either during this pregnancy or subsequently. The results indicated that patients with CHMCF may achieve live births after follow-up or adequate treatment.

Although not significant, we observed a trend indicating a higher incidence of subsequent GTN following CHMCF that necessitated infertility treatment (83% with GTN and 63% without GTN). Infertility treatments, such as ovarian stimulation, may elevate the incidence of CHMCF due to the increased likelihood of multiple births. However, numerous reports indicate no association between fertility treatment and molar pregnancy [14, 15]. The risk factors for developing post-molar GTN include being 40 years of age or older, having pre-curettage hCG exceeding 100,000 mIU/ml, and the presence of lutein cysts greater than 6 cm; however, infertility treatment is not regarded as a risk factor [1, 2]. The relationship between infertility treatment, CHMCF, and subsequent diseases remains controversial; therefore, further case studies are warranted.

This study has some limitations, particularly the small sample size of 14 patients. The single-center design limits the generalizability of the results across different institutions. The mono-ethnicity of the patients, being exclusively Japanese, suggests that the findings may not be generalizable to individuals of other ethnic backgrounds. The retrospective design may have introduced bias/obscured some results. However, these cases were reported by a single institution, indicating that the strategies for diagnosis, treatment, and follow-up were unified. Furthermore, the follow-up period exceeding four years constituted a significant advantage of this study.

In conclusion, the rate of GTN followed CHMCF at our institution was higher than that observed with typical complete or partial hydatidiform moles. Furthermore, patients diagnosed with GTN exhibited a greater likelihood of experiencing premature birth; however, our findings suggest that patients with CHMCF who choose to continue the pregnancies may still achieve live births. Consequently, obtaining informed consent from patients with CHMCF and their families is necessary for the continuation of pregnancy. Finally, collaboration among gynecological oncologists, obstetricians, and neonatologists is crucial for the effective diagnosis and treatment of CHMCF.
